# Mindfulness-Based Interventions for Survivors of Lung Cancer and Their Partners: A Systematic Review

**DOI:** 10.1007/s12529-022-10132-3

**Published:** 2022-10-12

**Authors:** Karen Kane McDonnell, Otis L. Owens, Fattona Umari

**Affiliations:** 1 Cancer Survivorship Research Center, College of Nursing, University of South Carolina, 1601 Greene Street, Columbia, SC 29208-4001 USA; 2 Healthy Aging Research and Technology (HART) Lab, College of Social Work; Core Faculty, Statewide Cancer Prevention and Control Program, Arnold School of Public Health, University of South Carolina, Columbia, SC USA; 3 College of Nursing, University of South Carolina, Columbia, SC USA

**Keywords:** Lung neoplasm, Dyads, Mindfulness-based interventions, Systematic review

## Abstract

**Background:**

Survivors of lung cancer and their partners often have complex unresolved physical, psychosocial, and behavioral needs that can negatively affect the survivors’ and partners’ well-being. This systematic review aimed to (1) examine the content and delivery of mindfulness-based interventions (MBIs) and (2) summarize and synthesize the current evidence for effectiveness of MBIs targeting survivors of lung cancer and/or one selected partner (dyads).

**Method:**

Six databases were searched for interventional studies published in English between 1980 and June 2020 using three terms (lung neoplasms, mindfulness, caregivers). For outcome measures, the interventions focused on behavioral change (meditation, yoga, stretching, breathing), symptom management (dyspnea, fatigue, sleep disruption, anxiety, depression, stress reduction), and knowledge. Two reviewers independently assessed article eligibility. One reviewer performed and another independently verified data extraction. The Cochrane risk-of-bias tool for randomized trials was used to critically appraise RCTs.

**Results:**

Searching yielded 307 records, of which 64 were assessed for eligibility. Six studies investigated the impact of an MBI on survivors and partners. Four studies were single-arm feasibility studies; two were RCTs. Two feasibility studies and one RCT recruited romantic couples whereas the others recruited asymmetrical dyads. The single-arm studies reported strong feasibility and acceptability. RCTs reported significant outcomes for reduced cancer-related distress and depression, and improved QOL, self-compassion, mindfulness skills, and rumination.

**Conclusion:**

Dyadic intervention research is a growing field. Few interventions target individuals with lung cancer and their partners. No interventions target partners alone. Future research should evaluate rigorous methodologies that enhance the understanding of independent and interdependent health-related effects within dyads and across relationships and settings.

**Supplementary Information:**

The online version contains supplementary material available at 10.1007/s12529-022-10132-3.

## Introduction

Lung cancer survivorship statistics are improving as overall mortality from lung cancer declines. In particular, the non-small cell lung cancer (NSCLC) mortality rate decreased substantially in the US population between 2013 and 2016, and mortality from other types of lung cancer, such as small cell lung cancer (SCLC), also decreased though to a lesser extent. The decline in death from NSCLC likely can be explained by a reduction in NSCLC incidence as well as advances in treatment, including introduction of targeted therapies [1]. With advances in life-prolonging treatments, more survivors are living longer. However, most survivors of lung cancer have undergone multiple invasive treatments and have a higher smoking-related chronic comorbidity burden, such as pulmonary disease (COPD) and heart disease, than survivors of other cancers [2].

Survivors experience a complex symptom burden pattern that has debilitating effects on their physical and psychological functioning and may seriously compromise their overall quality of life (QOL). The most common symptoms include dyspnea, fatigue, pain, sleep disturbances, emotional distress, depression, and anxiety. For survivors of lung cancer, the extent of the resection and other treatment modalities, unresolved treatment effects, comorbid conditions, smoking status, physical activity level, and emotional problems can further exacerbate symptoms [3]. The effect that stigma has on lung cancer survivorship may further complicate physical burdens, psychological distresses, and social challenges [4]. The strong association between a survivor’s emotional problems and debilitating symptom burden may impact treatment adherence, hospital readmission rates, and QOL [5]. These long-lasting effects must be important considerations regarding survivorship care interventions.

Family members, friends, and caregivers of survivors of lung cancer (hereafter referred to as “partners”) are also challenged by the diagnosis of lung cancer. They must cope with its psychological impact on the survivor, themselves, and others [6, 7]. Survivors and partners both react to serious illness and have a genuine need for help from healthcare providers [8, 9]. Partners provide emotional and practical support and must cope with their own health concerns, as well as the uncertainty surrounding the course of the illness and fear of losing their partner [5]. More than half of partners of lung cancer survivors report negative emotional effects of caregiving. An emerging consensus in the literature is that when the survivor and partner are treated simultaneously, the well-being of each individual improves [10]. When their needs are not addressed, partners are at risk for impaired mental and physical health. Despite this reality, most interventions have focused on the individual behavior of survivors only. However, the past decade has seen a growth in dyadic interventions exploring the effects of survivors and their partners (and their behaviors) on each other when a serious illness is diagnosed in one of them [11]. Recent studies found that survivors of lung cancer and their partners are interested in joint health promotion interventions [8, 9, 12]. Although survivors of lung cancer may be challenging to engage because of symptom burden, poor prognosis, or stigma, a great need exists for interventions to maximize QOL for both them and their partners. A greater emphasis on dyad-focused interventions is needed to better serve this population.

### Mindfulness and Cancer Care

A Cochrane review emphasized growing evidence of the positive value of mindfulness-based interventions (MBIs) for individuals with clinical health problems [13]. MBIs are described as promising in the management of cancer-related symptoms and treatment side effects [13–17]. In a 2020 meta-analysis by Xunlin and colleagues, the researchers showed that survivors with cancer who received MBIs reported significantly lower anxiety, depression, fatigue, and stress along with greater QOL, posttraumatic growth, and mindfulness than participants in a control group. After reviewing 26 RCTs, the authors concluded that MBIs were effective across different cancer types. Their summary indicated that most studies had either female participants only (*n* = 16) or both genders (*n* = 12); also, studies involved patients with breast cancer (*n* = 15), prostate cancer (*n* = 2), colorectal cancer (*n* = 2), lung cancer (*n* = 1), and a mix of cancers (*n* = 9) [15].

**Fig. 1:**
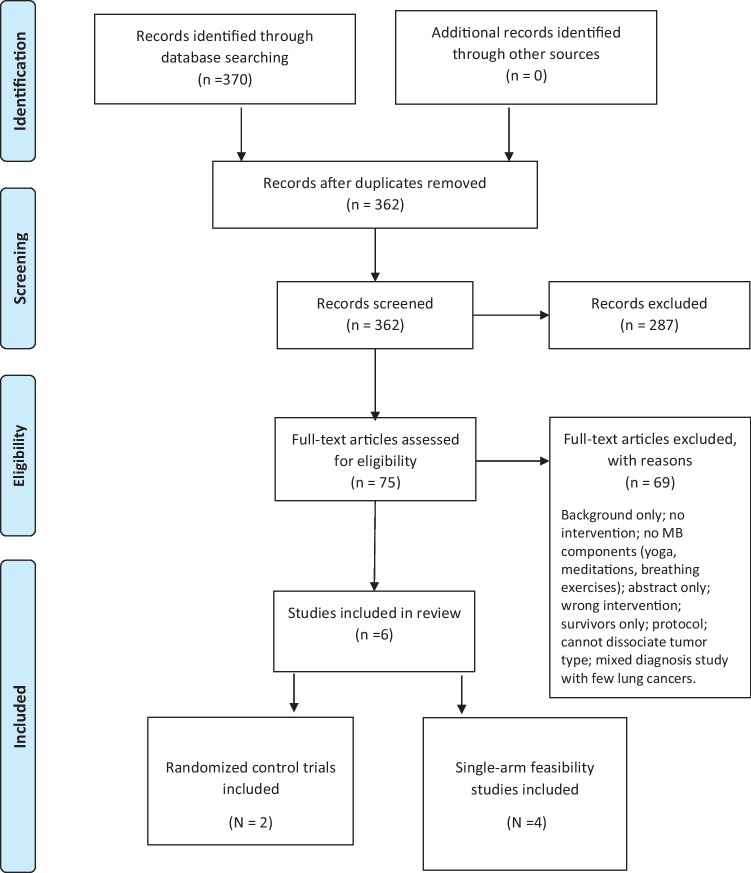
PRISMA Flow Diagram

MBIs incorporate breathing practices, meditations, and yoga; target self-management of challenging emotional and physical symptoms; and may provide an effective approach to symptom management [17]. Over 10 years ago, MBIs were found to be feasible and acceptable in some populations with cancer, as well as effective at reducing psychological stress [18]. However, the generalization of results with various populations of cancer survivors is limited because gaps exist: most participants in the MBIs were female survivors with breast cancer. This systematic review emphasizes that only a few MBIs have focused on the specific needs of survivors of lung cancer and their partners (see Fig. 1). This systematic review aimed to (1) examine the content and delivery of MBIs targeting survivors of lung cancer and/or their partners (dyads), and (2) summarize and synthesize the evidence for feasibility and efficacy of these interventions.

## Methods

Guidance from the Centre for Reviews and Dissemination and from the Preferred Reporting Items for Systematic Reviews and Meta-Analyses (PRISMA) informed the methods for conducting and reporting this review [19]. With the assistance of a research librarian at the University of South Carolina, six electronic databases were searched using terms related to three main concepts: lung neoplasms, mindfulness, and caregivers. Keywords related to the three concepts were combined with an “OR” Boolean operator and eventually combined with the subject headings by using the “AND” operator. This process was performed across each of the six databases: PubMed, Web of Science, CINAHL Complete, PsycINFO, Embase, and Cochrane CENTRAL. Search strategies were predefined and created by two investigators in collaboration with a librarian. The same search across all six electronic databases was performed a second time by a graduate research assistant to ensure that no errors were made the first time (see Appendix ESM for the full search strategy).

### Study Selection (Inclusion and Exclusion Criteria)

To be included in this review, studies were required to (1) include an MBI; (2) be conducted with survivors of lung cancer and/or their partners, aged 18 or older; (3) be published between January 1980 and June 2021; and (4) be written in English. Survivors of lung cancer were defined as individuals who had had a lung cancer diagnosis. Partners were defined as individuals who served in informal or formal social support roles to the survivor. Studies targeting the following outcomes were prioritized for this review: behavioral change (meditation, yoga, stretching, and breathing), symptom management (dyspnea, fatigue, sleep disruption, anxiety, depression, and stress reduction), physical function (exercise capacity), QOL, and knowledge. Studies of any type or design (qualitative/quantitative, systematic reviews, or RCTs) were included in this review. Studies focusing on multiple cancers met the inclusion criteria only if they analyzed data separately for lung cancer. Studies were excluded if they met any one of the following criteria: (1) involved interventions unrelated to mindfulness; (2) did not include survivors of lung cancer and/or partners; (3) were not available in English; (4) were editorials, commentaries, or study protocols; (5) had outcome measures outside those prioritized in the inclusion criteria (e.g., drug interventions, biomarkers, genetic testing/evaluation).

### Data Extraction

Following the searches of the six databases, resulting articles were initially uploaded to EndNote for removal of duplicate articles. Next, the remaining articles were transferred from EndNote to Covidence, an online software program that facilitates a multiperson, multistep article review process of the titles, abstracts, and full text of articles [20]. Specifically, two reviewers independently selected studies for inclusion using a two-step process. First, studies underwent screening based solely on the title and abstract. In this step, each reviewer compared the title and abstract against the review criteria to ascertain whether the article would continue to a full-text review. When the two reviewers disagreed (in Covidence) whether an article should be considered for inclusion in this systematic review, they resolved their disagreement during a discussion session. Articles that met the inclusion criteria based on the title or abstract were downloaded to a shared folder and uploaded to Covidence for a second review round. The second stage of review mirrored the first. Following the second-stage review, the final set of articles was downloaded to a shared folder where the authors conducted further investigation about the effectiveness of MBIs for survivors of lung cancer and/or their caregivers.

### Risk of Bias

The Cochrane Risk of Bias Tool (RoB 2) for RCTs was used to assess bias in the two RCTs [21]. This tool is structured into a fixed set of required domains of bias focusing on distinct aspects of trial design, conduct, and reporting (randomization process, deviations from intended interventions, missing outcome data, measurement of outcomes, and selection of the reported results). RoB 2 is comprised of a series of “signaling questions,” which lead the reviewers to make a judgement about the risk of bias for a particular domain, followed by a justification of the judgement, and a prediction of the likely direction of the bias.

## Results

A total of six studies, all investigating the impact of MBIs on survivors of lung cancer and partners, were included in the review (see Tables 1 and 2). No studies were found that targeted partners alone. Four were single-arm feasibility studies [22–25] with sample sizes ranging from 14 participants (7 dyads) to 62 participants (31 dyads). Three reviewed studies were pilot studies using a mixed-method design [22, 24, 25]. Two studies were RCTs [26, 27] with sample sizes ranging from 107 participants (41 dyads; some participants were not part of a dyad) to 150 participants (75 dyads) distributed among three groups. The smaller RCT compared mindfulness-based stress reduction (MBSR) plus usual care (UC) to UC alone. The larger RCT had three arms comparing a couple-based meditation (CBM) intervention to a supportive-expressive intervention and UC. Studies were conducted in the Netherlands or the USA. Unlike most previous studies conducted with survivors of cancer, all reviewed studies targeted both genders. Only studies conducted in the USA reported on racial or ethnic diversity.

**Table 1:** Mindfulness-based interventions for dyads: feasibility results

Reference	Cancer type, stage, and treatment phase	Setting, design, sample size, and sample characteristics	Intervention description	Outcomes and measures	Major findings
Single-arm studies					
McDonnell et al. [22]	• NSCLC • Stages I, II, IIIA • Partner: family member or close friend • Time since DX: within 12 y • Survivorship phase (not undergoing treatment)	• USA, urban community, 2-month prospective, 1-group, pre/post design *• N* = 62 • Gender: SXs, 38% male; partners, 57% male • Age, y (± SD): SXs, 66.5 (5.5); partners, 60.2 (14.1) • Race: SXs, 65% Black; partners, 61% Black; all others White • COPD: SXs, 46%; partners, 4% • Current smokers: SXs, 8%; partners, 13%	• Based on MBSR • 10 wk, 12 in-person sessions (90 min each) • Included meditation; two yoga levels (level I, chair yoga; level II, standing and floor yoga); breathing exercises; education on dyspnea, physical activity and fatigue, insomnia, body balance, responding to stress, communication, and mindfulness • Manuals and trac phones (used as MP3 players) provided for home use • Weekly home assignment logs • Instructors were qualified mindfulness trainers	• Outcomes: Recruitment, Retention, Intervention dose, Adherence, Acceptability • Measures: Dyadic qualitative interviews, exploring feasibility; MBSR experiences (post-test)	• Feasibility: safe and acceptable • Recruitment: SXs, 20% • Retention: SXs, 81%; partners, 78% • Adherence: exceeded attendance expectations, moderately strong at-home practices (5 days/wk) • Acceptability: strong positive ratings (range, 80–100%) for 10 indicators
Milbury et al. [23]	• NSCLC • Stages I, II, or IIIB • Partners: family caregivers (e.g., spouse, sibling) • Time since DX (± SD): 2.9 mo (1.4) Treatment phase (undergoing RT)	• USA, urban academic cancer center, single-arm trial *• N* = 20 • Gender: SXs, 50% male; partners, 10% male • Age, y (± SD): SXs, 71.2 (6.2); partners, 68.7 (6.0) • Race: 80% White, 10% Latino/Hispanic, 10% Other • COPD: SXs, 60%; • Current smokers: not reported	• 5–6 wk, 15 in-person sessions (45–60 min each) • Included deep breathing awareness with visualization, breath retention exercises, guided meditations, seated Tsa Lung movements, and compassion • Received program CD and printed materials • Instructors were yoga trainers	• Outcomes: • Consent rates, attendance, assessment completion, program evaluation	• Feasibility: safe and acceptable • Consent rate: dyads 74% • Retention: 71% • Attendance: 80% of dyads attended more than 50% of all sessions • Evaluation: 92% rated “useful or very useful”
Milbury et al. [24]	• NSCLC • Stage IV • PARTs: romantic partners • Time since DX (± SD): 4.17 mo (3.35) • Treatment phase: 100%	• USA, urban academic cancer center, single-arm trial *• N* = 12 • Gender: SXs, 33% male; partners, 67% male • Age, y (± SD): SXs, 55.2 (5.16); partners, 59.4 (5.71) • Race: 100% White (non-Hispanic) • COPD: not reported • Current smokers: SXs, 33%; partners, 33%	• 2 wk, 4 in-person sessions (60 min each) • Included mindful breathing exercises and compassion-based meditations • 4 topic areas: Mindfulness, Connection, Gratitude, Purpose • CDs and printed materials were provided for home use • Instructors: not reported	• Outcomes: Consent rate, attendance, home practice completion, acceptability, usefulness, perceived benefit, perceived difficulty	• Feasibility: • Consent rate: 54% • Retention rate: 57% • Homework completion: 100% Viewed as beneficial: 38% • Highly acceptable, perceived as “useful or very useful” • Greatly benefitted 75%
van den Hurk et al. [25]	• Mixed cancer type: NSCLC, 79%; SCLC, 21% • Stages II, III, IV • Partners: Type not specified • Time since DX (range): 8.4 mo (2–35) • Treatment phase: 74%	• Netherlands, tertiary academic medical center, single-arm trial, • mixed methods, pre/post, 3-month design *• N* = 35 • SXs, *n* = 19; partners, *n* = 16 • Gender: SXs, 53% male; partners, 44% male • Race: No minorities reported • Age, y (range): SXs, 61.7 (54–77); partners, 60.9 (30–76) • COPD: not reported • Current smokers: not reported	• Based on MBSR • 8 in-person sessions, 2.5 h each, with an additional silent day, and home assignments of 45 min/day • CD and workbook provided; psycho-education about grief • Instructors were qualified mindfulness trainers	• Outcomes: Facilitators, barriers, processes of personal change Measures: Qualitative interviews (optional) explored feasibility and MBSR experiences within a year post intervention completion, (SXs, *n* = 6; partners, *n* = 5)	• Feasibility: • Retention: post, SXs, 68%; partners, 69%; • 3-month follow-up, SXs, 47%; partners, 50% • Interview themes included: facilitators and barriers; and processes of change
Randomized controlled trials (RCTs)					
Milbury et al. [26]	• NSCLC • Stage IV • Partners were romantic partners • Time since DX (± SD): CBM, 53.0 wk (54.6); SE, 44.3 wk (54.7); UC, 68.4 wk (96.5) • Treatment phase: 100%	• USA, academic cancer center, 3-arm pilot RCT; CBM, SE, UC *• N* = 150 • SXs, *n* = 75; partners, *n* = 75 • Dyad assignment: CBM, *n* = 26; SE, *n* = 24; UC, *n* = 25 • Gender: SXs—CBM, 50% male; SE, 50% male; UC, 48% male partners—CBM, 42% male; SE, 50% male; UC, 52% • Race: SXs, 85% White (non-Hispanic); partners, 79% White (non-Hispanic) • Age (± SD): SXs, 65 yr (10.4); partners, 63.9 yr (10.3) • COPD: not reported • Current smokers: not reported; never smokers: SXs, 52%; partners, 35%	• 4 wk, 4 sessions, 60 min each, delivered by videoconference (FaceTime) one-on-one with facilitator; reassessed at 1- and 3-mo post-intervention • Used CBM (rooted in the constructs of interconnectedness, mindfulness, and compassion) • Audio files of the meditations and printed materials of the session content were provided • Instructors were master’s-level facilitators (psychology counselor interns)	• Outcomes: Consent rates, Study attrition, Session attendance, Homework completion, Session content satisfaction, Dyadic and program delivery, Overall usefulness, benefit	• Feasibility: • Consent rate: 63%; • Retention: 65%; • Attendance: 71%, attendance did not vary by group; • Benefit: CBM rated intervention sig. more beneficial; • Delivery preference: dyad • Format preference: online (38% SX, 35% partners; no preference (38% SXs, 41% partners); in-person (25% SXs, 24% partners)
Schellekens et al. [27]	• Mixed cancer type: 86%; NSCLC, 11%; SCLC, 3%; mesothelioma; • Stages I, II, III, IV • Partners: life partners, family members, friends • Time since DX (SD): IG, 4.8 mo (4.5); UC: 9.3 mo (10.8) • Treatment phase: undergoing CT, RT, or both; Survivorship phase: (not undergoing treatment)	• Netherlands, multicenter, 2-group, parallel-group RCT; MBSR + CAU (IG), CAU (MILON study) *• N* = 107; IG: *n* = 52 (SXs, *n* = 31; partners, *n* = 21); CAU: *n* = 55 (SXs, *n* = 32; partners, *n* = 23) • Gender: SXs—IG, 42% male; CAU, 53% male; partners: IG, 43% male; CAU, 53% male • No minorities, COPD or smoking status reported	• Based on MBSR • 8 wk, in-person small groups (9 groups total using the intervention); 2.5-h sessions + one 6-h silent retreat, including (a 45-min daily home practice); UC included anticancer treatment(s), medical consultations, and supportive care including psychosocial care • Instructors were formally trained MBSR teachers	• Outcomes: No feasibility outcomes indicated	• Recruitment: 18% • Participation with a partner: 65% • Retention: 81%

“Partner” refers to a partner (spouse), a family member, or a caregiver (informal or formal support role)

*NSCLC*, non-small cell lung cancer, *DX* diagnosis, *SX* survivor of lung cancer, *IG* intervention group, *CBM* couple-based meditation group, *UC/CAU* usual care, *MBSR* mindfulness-based stress reduction, *RT* radiation therapy, *CT* chemotherapy

**Table 2:** Mindfulness-based interventions for dyads and partners: preliminary effects results

Reference	Preliminary effects	Measures	Major findings
Single-arm studies			
McDonnell et al. [22]	Dyspnea, fatigue, stress, sleep disturbance, anxiety, depression, exercise tolerance	FACIT–D, FACIT–F, PSS, PSQI, HADS, 6MWT	• Survivors experienced greater improvement in symptom scores than partners • Level I participants benefited most. Reported significant less dyspnea (*p* = 0.093), with survivors experiencing greater benefit (*p* = 0.075) post-test. All participants reported significant improvement in fatigue (*p* = 0.075). Demonstrated significantly improved exercise capacity (survivors, *p* = 0.09; partners, *p* = 0.05)
Milbury et al. [23]	Anxiety, psychological distress, sleep disturbances, fatigue	BSI 18, BFI, CES-D, PSQI, SF-36, FACT, Finding Meaning in Cancer Scale	• Survivors reported significant improvements in spiritual well-being (*t* = 30, *p* = 0.03, *d* = 1.12) and a marginally significant in benefit finding (*t* = 2.0, *p* = 0.08, *d* = .71). There were also medium effect sizes for depression symptoms (*d* = 0.52) and sleep disturbances (*d* = 0.60) and a small effect for anxiety and Mental Health Component Scale (MCS scores) • Partners reported significantly less fatigue (*t* = 2.7, *p* = 0.03; *d* = 0.89) and anxiety (*t* = 2.4, *p* = 0.04, *d* = 0.81) and a marginally significantly reduction in sleep disturbance (*t* = 2.0, *p* = 0.08, *d* = 0.7). Medium effect sizes were found for improved benefit finding (*d* = 0.52) and small effect sizes for depressive symptoms and MCS scores
Milbury et al. [24]	Spirituality, psychological distress, impact of events, sleep disturbances	FACT-Sp, CES-D, IES, PSQI	• Survivors reported reduced sleep disturbances (large effect, *P* = 0.04, *d* = 1.83), less cancer-specific distress (medium effect, *d* = 0.61), improvements in depressive symptoms (small effect, *d* = 0.29), and improved spiritual well-being (small effect, *d* = 0.29) • Partners reported reduced depressive symptoms (large effect, *d* = 0.90), increased sleep disturbances (small effect, *d* = 0.27), with no meaningful differences for psychological distress or spiritual wellbeing
van den Hurk [25]	Psychological distress, QOL (dyspnea, hemoptysis, coughing, pain), fatigue, psychological stress reaction, lapses of attention/awareness, caregiver appraisal, care-derived self-esteem	NCCN DT, HADS, CIS-F, CRA-SE, EORTC QLQ-LC13, IES, PSWQ, MAAS, SPPIC	• No significant differences found in pulmonary symptomology, fatigue, pain, anxiety, depression, mindfulness skills, or worry. Mean scores for anxiety and depression decreased post-intervention for survivors and partners, the change was not significant. The extent to which caregiving was experienced as burdensome by partners decreased significantly at post-intervention and at 3 mo. follow-up
Randomized controlled trials (RCTs)			
Milbury et al. [26]	Depression, cancer-related stress, spiritual well-being	CES-D, IES, FACT-Sp	• The group main effect for depression in survivors was not significant (*F* = 0.05, *p* = .95). Partners experienced a significant group main effect (*F* = 3.70, *p* < .05; least square means between three groups: CBM = 6.73. SE = 9.32, UC = 11.57) • No significant group differences were reported for survivors’ cancer-related stress scores (*F* = 0.16, *p* = .85; least square means: CBM = 15.48, SE = 16.66, UC = 17.17). No significant group differences were reported for partners’ cancer-related stress scores (*F* = 0.32, *p* = .73; least square means: CBM = 17.76, SE = 17.32, UC = 19.60) • No significant group differences were reported for survivors’ (*F* = 0.58, *p* = .57) or partners’ (*F* = 1.79, *p* = .17) spiritual well-being
Schellekens et al. [27]	Psychological distress, QOL, caregiver burden, relationship satisfaction, mindfulness skills, self-compassion, rumination, post-traumatic stress symptoms	HADS, EORTC QLQ-LC13, IMS-S, SPPIC, FFMQ, SCS, RRS-B, IES	• Using intent-to-treat analyses, the intervention group (IG) survivors showed significantly less psychological distress post-intervention and at follow-up (*p* = .008, *d* = .69), and reported less anxiety (*p* = .007, *d* = .620 and depression (*p* = .027, *d* = .69) when compared to non-IG survivors • Significant improvement in IG survivors’ QOL (*p* = .047, *d* = .60), mindfulness skills (*p* = .001, *d* = .84), self-compassion (*p* = .009, *d* = .80), and rumination (*p* = .018, *d* = .67). A trend showed improvement in posttraumatic stress symptoms (*p* = .051, *d* = .37) • Intent-to-treat analysis did not show any differences at post-intervention or follow-up between IG partners and non-IG partners. A trend showed a decrease in relationship satisfaction in the IG group post-intervention (*p* = .055, *d* = .63)

“Partner” refers to a partner, family member, caregiver, or close friend in a formal or informal support role to the survivor

*Abbreviations of measures*: *FACIT–D* FACIT–Dyspnea Short Form, Part 1, *FACIT–F* FACIT Fatigue Scale, *PSS* Perceived Stress Scale, *PSQI* Pittsburgh Sleep Quality Index, *HADS* Hospital Anxiety and Depression Scale, *6MWT* Six-Minute Walk Test (measures exercise capacity), *BSI 18* Brief Symptom ,Inventory-18 (anxiety dimension), *BFI* Brief Fatigue Inventory, *CES-D* Center for Epidemiology Studies-Depression, *SF-36* 36-Item Short Form Health Survey (RAND Medical Outcomes Study), *FACT-Sp* Functional Assessment of Cancer Therapy Spirituality and Wellbeing Scale, *IES* Impact of Events Scale, *NCCN DT* National Comprehensive Cancer Network Distress Thermometer, *CIS-F* Checklist Individual Strength, *CRA-SE* Caregiver Reaction Assessment (Self-Esteem subscale), *EORTC QLQ-LC13* European Organization for Research and Treatment of Cancer Core Quality of Life Questionnaire for Lung Cancer, *PSWQ* Penn State Worry Questionnaire, *MAAS* Mindful Attention Awareness Scale, *SPPIC* Self-Perceived Pressure from Informal Care, *MDASI–LC* MD Anderson Symptom Inventory (for lung cancer), *SCS* Self-Compassion Scale, *ECR-S* Experiences in Close Relationship Scale (Short Form), *IMS-S* Investment Model Scale (satisfaction construct), *FFMQ* Five Facet Mindfulness Questionnaire, *RRS-B* Ruminative Response Scale (brooding subscale)

### Content and Delivery

Most interventions were conducted in person with at least weekly sessions and lasted 2 to 8 weeks; sessions lasted between 45 and 150 min and included recommended home assignments. One of the RCTs used videoconferencing (FaceTime) as the delivery method in a palliative care setting [26], whereas the other RCT took place in a multisite cancer center setting [27]. The RCTs conducted follow-up at 1 and 3 months or at 3 months post-intervention [26, 27].

The content varied by study. Three interventions were modeled after an MBSR approach [22, 25, 27]. Content areas included symptom management, physical activity and body mechanics, grief, stress management, communication, and mindfulness. Participants received instructions on a variety of formal and informal practices, including meditations, breathing practices, stretching, and yoga. Three other interventions with the same primary author (two pilots, one RCT) had varied approaches and content [23–25]. The earliest pilot intervention [23] included a type of Tibetan yoga known as Tsa Lung, which focuses on movements directed to the upper body. Breathing exercises, gentle movements, guided visualizations, and emotional connectedness were included. The second pilot [24] focused a brief intervention on cultivating mindfulness, interpersonal connection, gratitude, and a sense of personal purpose. The third RCT [25] used a mindfulness approach to management of couples’ psychospiritual distress.

### Recruitment and Retention

All studies targeted survivors with lung cancer. Studies enrolled individuals with early-stage disease [22] or any stages [23, 25, 27]. Two studies enrolled individuals with stage IV or advanced lung cancer only [24, 26]. Survivors ranged from newly diagnosed (within months of diagnosis) and undergoing treatment to longer-term survivors (up to 12 years post-diagnosis) [22, 23]. Most studies enrolled patients with NSCLC. One study included participants with SCLC and mesothelioma [26]. In terms of partners, two studies recruited “romantic couples” [22, 23]. Other dyadic studies recruited family members only (e.g., spouses or siblings) or asymmetric partners (whomever the patient described as supportive—all were family members or close friends). Three studies reported recruitment rates of 18%, 20%, and 74% [22, 23, 27]. Several reported retention rates. The highest retention rates (> 70%) were reported for the studies that enrolled survivors with stages I–IIIB lung cancer [22, 23].

Unfortunately, one of the greatest challenges to interventions targeting individuals with advanced or metastatic disease is mortality during or after the intervention. Five out of six studies reported participants’ deaths during the study or post-intervention [24–27] or reported that two partners were diagnosed with lung cancer during the intervention [22]. One was diagnosed with early-stage NSCLC and survived. The other, diagnosed with advanced-stage NSCLC, died a few months after the intervention was completed.

### Participant Characteristics

Gender diversity was represented in these studies. Recruitment of male survivors ranged from 33 to 53% across studies. In terms of partners, male representation ranged from 10 to 67%. All studies recruited survivors who were older adults. Partners tended to be younger than their survivor counterparts. Race and ethnicity were reported in the studies conducted in the USA (*n* = 4). Regarding the US studies that reported race/ethnicity, one study reported an enrollment of 65% Black survivors and 61% Black partners [22]. Three other studies reported no or few minorities [23, 24, 26]. Half of the studies reported smoking status.

### Evidence for Feasibility and Efficacy

Good to strong feasibility and acceptability was reported by four pilot studies conducted with survivors with local, advanced, and metastatic disease as well as patients undergoing active treatment (see Table 1). In terms of preliminary outcomes, pilot studies suggested reduced dyspnea, fatigue, sleep disturbances, and emotional distress, plus improved functional exercise capacity and spiritual well-being, for survivors. Reductions in anxiety, depression, caregiver burden, and fatigue were reported by partners. The RCTs suggested that psychological distress may be treated effectively with MBSR interventions, and that MBIs may help with managing psychological symptoms in the palliative care setting (see Table 2).

### Bias Assessment

Two reviewers examined the RCT protocols [28, 29] and journal articles with trial results for bias. Both RCTs were judged to be at some risk of bias in at least one domain. Milbury et al. [28] acknowledged that the sample size was not determined by a power calculation because the study was not designed to determine efficacy (p. 4). The randomization process is described as “minimization” to ensure that the three groups were balanced according to four prognostic factors (age, sex, Eastern Cooperative Oncology Group [ECOG] performance status, and National Comprehensive Cancer Network [NCCN] distress score). In the study’s protocol, the authors describe the trial as “unmasked” to participants and care providers, but the research staff members involved in data collection were blind to the group allocation [28]. No baseline imbalances in the prognostic characteristics existed to suggest a randomization mishap, although distress score results were not reported. Neither was there any information on co-interventions or protocol deviations. A 35% attrition rate was realized, with death being the major reason for attrition. No differential dropout was reported based on group assignment. An intent-to-treat analysis and the PROC MIXED statement in SAS 9.4 were used to manage missing data. Insufficient information is available to rule out the possibility that reported outcomes were selected, based on the results, from multiple outcomes measures.

In Schellekens et al. [29], randomization was stratified by site and minimized by four factors: stage of disease, baseline HADS (Hospital Anxiety and Depression Scale) score, treatment phase (no treatment versus treatment with chemotherapy and/or radiation therapy), or participation as a single or dyad. No information was provided regarding allocation sequence randomization. Baseline differences between survivors in each study arm included months since diagnosis and current treatment. No baseline differences existed between partners assigned to either study arm, and no baseline differences existed between completers and noncompleters of the MBSR study arm. No information was provided regarding co-interventions. Protocol deviations may have been related to the MBSR teachers, tumor type, and sample size. One of three formally trained MBSR teachers (described as beginner level) left the study. The authors asserted that different teachers did not affect participant outcomes. Teacher effectiveness was monitored throughout the study. Even though study duration was extended to maximize enrollment, sample size was still smaller than anticipated. Original power analysis used G*Power (and an anticipated 20% dropout rate) to determine that 110 survivors and 110 partners were required [29]. Instead, only 107 participants total enrolled (62 survivors and 55 partners; 41 survivors [66%] participated with a partner). Missing data (25–29%) was attributed to dropouts, which were in turn attributed to study burden, lack of study appeal, or patient death. No missing-data management plan was identified. Intent-to-treat analysis was used. Results were reported for the primary outcome and six secondary outcomes.

## Discussion

The aims of this systematic review were to examine the content and delivery of MBIs targeting survivors of lung cancer and partners (dyads) or their partners alone, and to synthesize the evidence for feasibility and efficacy of these interventions. No studies were found targeting partners alone, and few studies involved both survivors and partners.

The feasibility and acceptability data confirmed interest in MBIs among survivors and their partners [22–27]. In terms of adherence, participation of individuals receiving active cancer treatment varied when compared against individuals not being treated. For the long-term survivors’ group (those not in active treatment), intervention attendance was greater than 90% for both survivors and partners, and the intervention completion rate was 81% [22]. For studies targeting survivors with advanced lung cancer undergoing treatment, the retention rates were lower (approximately 70% on average). This result is similar for other MBI studies targeting other types of cancer survivors. For example, a similar MBI study involving survivors of advanced-stage solid tumor cancers and partners reported a retention rate of about 70% (across six intervention sessions) and retention rates of 84% and 92% for survivors and partners, respectively. For survivors undergoing treatment, attrition was explained mostly by physical impairments caused by either illness progression or anti-cancer treatments [30]. This attrition rate was similar to those in studies in this review [24, 26, 27].

All studies enrolled both male and female survivors. No subgroup analyses by gender were reported. In a systematic review and meta-analysis of 29 RCTs (*N* = 3476 survivors), subgroup analyses of gender differences were reported that led the authors to suggest that “MBIs might be potentially more effective in women compared to men” ([15], p. 1577). However, Ford, Vowles, Smith, & Kinney (2020) examined the effects of meditative movement interventions and MBIs on men with cancer in 17 RCTs (*N* = 666 survivors) and concluded that evidence exists for improving psychosocial outcomes post-intervention. The authors acknowledged the need to design studies with active control groups and greater short-term and long-term follow-up assessments beyond the immediate intervention period. The importance of measuring continued practice post-intervention is a key factor in sustainability of effect [31].

More rigorous efficacy studies are needed. In this review, the Cochrane RoB 2 was used to assess bias in the two RCTs [21]. One RCT was described as not powered [26]. The other RCT recruited mixed tumor types [27]. Overall, both studies were judged to be at some risk of bias in at least one domain.

One study in this review evaluated the hybrid delivery of a dyadic intervention through videoconferencing in a palliative care setting. The recruitment and retention rates were 63% and 64%, respectively. Delivery preferences were assessed. The preference breakdowns were online—38% survivors, 35% partners; in person—25% survivors, 24% partners; and no preference—38% survivors, 41% partners [26]. In an RCT of a dyadic psychosocial intervention (non-MBI) administered by telephone to survivors with advanced lung cancer and their partners (dyads), the recruitment rate was 60%, and retention was rated “excellent” [32]. Knowing the numbers of individuals with advanced lung cancer and their symptom burden, remote intervention delivery might be an acceptable option for this population. The use of technology may allow more survivors with advanced lung cancer and their partners to participate in clinical research studies. More research is needed to assess preferences for intervention delivery options and factors that act as facilitators and barriers to participation. Testing flexible delivery options (mobile health, Internet) to allow for efficient delivery and widespread dissemination is needed.

Partners of survivors of lung cancer also must cope with its physical and psychological impact on the survivor, themselves, and others. It can be assumed that dyadic interventions recognize that survivors and their partners may react to a serious illness and as a result they both have a genuine need for help from healthcare providers. Partners not only provide emotional and practical support, but they must also cope with their own physical, emotional, and social concerns, including the uncertainty surrounding the course of the illness, and fear of losing their partner [6, 7, 33]. More attention on the partners’ experience is needed so that a greater understanding of their needs is developed.

An emerging consensus in the literature is that when both the survivor and partners are treated simultaneously, the well-being of each individual may improve. When their needs are not addressed, partners are at risk for impaired health. However, the past decade has seen a growth in interventions exploring the effects of FMs (and their behaviors) on each other when a serious illness is diagnosed in one of them [5].

A greater emphasis is needed on interventions that target partners (caregivers, family members, and friends), not only the survivor.

A recent state-of-the-science paper on dyadic interventions for cancer survivors and caregivers identified new directions for dyadic intervention research. Inclusion of individuals with diverse minority backgrounds (race, ethnicity, and religious), lower socioeconomic status, diverse dyadic relationships (other than spouse), and various disease stages are needed [5]. Diversifying targeted populations will enhance understanding of the role of dyadic relationships in adaptation to lung cancer as a chronic disease and the importance and extent of cultural influences.

## Conclusions

The field of dyadic intervention research is growing, but major scientific gaps exist. Few studies have targeted survivors of lung cancer, and even fewer have targeted survivors of lung cancer together with partners (dyads) or partners alone. Overall, designing studies to enhance understanding of various independent and interdependent health-related effects within dyads across relationships and settings will benefit the science of dyadic supportive care interventions.

### Supplementary Information

Below is the link to the electronic supplementary material.

Supplementary file1 (DOCX 32 KB)
