# Supplementary material for: Mindfulness-Based Interventions for Survivors of Lung Cancer and Their Partners: A Systematic Review
Source: Int J Behav Med. 2022 Oct 12;30(5):616–27. doi: 10.1007/s12529-022-10132-3 (PMC10522728; doi:10.1007/s12529-022-10132-3)
Supplement: Supplementary file 1 — Supplementary file1 (DOCX 32 KB) [file 12529_2022_10132_MOESM1_ESM.docx]

| Appendix  *Search Strategy* | | | |
| --- | --- | --- | --- |
| Database | Concept 1:  Lung Neoplasms | Concept 2:  Mindfulness | Concept 3:  Caregivers |
| PubMed | 1. Subject heading: Lung Neoplasms [MeSH] | 1. Subject headings: Breathing Exercises [MeSH:NoExp] OR Meditation [MeSH] OR Mindfulness [MeSH] OR Stress, Psychological/psychology [MeSH] OR Stress, Psychological/therapy [MeSH] OR Yoga [MeSH] | 1. Subject headings: Adult Children [MeSH] OR Caregivers [MeSH] OR Family [MeSH:noexp] |
|  | 1. Keywords: (cancer of the lung* [tw] OR lung cancer* [tw] OR lung carcinoma* [tw] OR lung neoplasm* [tw] OR lung tumour* [tw] OR lung tumor* [tw] OR nsclc [tw] OR pulmonary carcinoma* [tw] OR pulmonary tumor* [tw] OR pulmonary tumour* [tw] OR sclc [tw]) | 1. Keywords: breathing exercis* [tw] OR MBCR [tw] OR MBCT [tw] OR MBSR [tw] OR meditat* [tw] OR mindful* [tw] OR stress reduc* [tw] OR yoga [tw] | 1. Keywords: adult children [tw] OR care giver* [tw] OR caregiver* [tw] OR care giving [tw] OR caregiving [tw] OR carer [tw] OR carers [tw] OR care taker* [tw] OR caretaker* [tw] OR caring [tw] OR couple* [tw] OR custodian* [tw] OR daughter* [tw] OR dependents [tw] OR dyad* [tw] OR families [tw] OR family [tw] OR folk* [tw] OR guardian* [tw] OR husband* [tw] OR kinship [tw] OR parent* [tw] OR partner* [tw] OR relatives [tw] OR sibling* [tw] OR son [tw] OR sons [tw] OR spous* [tw] OR wife [tw] OR wives [tw] |
| Web of Science | 1. Subject headings: n/a 2. Keywords: "cancer of the lung*" OR "lung cancer*" OR "lung carcinoma*" OR "lung neoplasm*" OR "lung tumour*" OR "lung tumor*" OR nsclc OR "pulmonary carcinoma*" OR "pulmonary tumor*" OR "pulmonary tumour*" OR sclc | 1. Subject headings: n/a 2. Keywords: "breathing exercis*" OR MBCR OR MBCT OR MBSR OR meditat* OR mindful* OR "stress reduc*" OR yoga | 1. Subject headings: n/a 2. Keywords: "adult children" OR "care giver*" OR "caregiver*" OR "care giving" OR "caregiving" OR carer OR carers OR "care taker*" OR "caretaker*" OR caring OR couple* OR custodian* OR daughter* OR dependents OR dyad* OR families OR family OR folk* OR guardian* OR husband* OR kinship OR parent* OR partner* OR relatives OR sibling* OR son OR sons OR spous* OR wife OR wives |
| CINAHL Complete | 1. Subject heading: (MH "Lung Neoplasms+") | 1. Subject headings: (MH "Breathing Exercises") OR (MH "Meditation") OR (MH "Mindfulness") OR (MH "Stress, Psychological/TH") OR (MH "Yoga+") | 1. Subject headings: (MH "Adult Children") OR (MH "Caregivers") OR (MH "Family") |
|  | 1. Keyword search (title only): "cancer of the lung*" OR "lung cancer*" OR "lung carcinoma*" OR "lung neoplasm*" OR "lung tumour*" OR "lung tumor*" OR nsclc OR "pulmonary carcinoma*" OR "pulmonary tumor*" OR "pulmonary tumour*" OR sclc | 1. Keyword search (title only): "breathing exercis*" OR MBCR OR MBCT OR MBSR OR meditat* OR mindful* OR "stress reduc*" OR yoga | 1. Keyword search (title only): "adult children" OR "care giver*" OR caregiver* OR "care giving" OR caregiving OR carer OR carers OR "care taker*" OR caretaker* OR caring OR couple* OR custodian* OR daughter* OR dependents OR dyad* OR families OR family OR folk* OR guardian* OR husband* OR kinship OR parent* OR partner* OR relatives OR sibling* OR son OR sons OR spous* OR wife OR wives |
|  | 1. Keyword search (abstract only): "cancer of the lung*" OR "lung cancer*" OR "lung carcinoma*" OR "lung neoplasm*" OR "lung tumour*" OR "lung tumor*" OR nsclc OR "pulmonary carcinoma*" OR "pulmonary tumor*" OR "pulmonary tumour*" OR sclc | 1. Keyword search (abstract only): "breathing exercis*" OR MBCR OR MBCT OR MBSR OR meditat* OR mindful* OR "stress reduc*" OR yoga | 1. Keyword search (abstract only): "adult children" OR "care giver*" OR caregiver* OR "care giving" OR caregiving OR carer OR carers OR "care taker*" OR caretaker* OR caring OR couple* OR custodian* OR daughter* OR dependents OR dyad* OR families OR family OR folk* OR guardian* OR husband* OR kinship OR parent* OR partner* OR relatives OR sibling* OR son OR sons OR spous* OR wife OR wives |
|  | 1. Combination search: Search 1 OR Search 2 OR Search 3 | 1. Combination search: Search 1 OR Search 2 OR Search 3 | 1. Combination search: Search 1 OR Search 2 OR Search 3 |
| PsycINFO | 1. Subject heading: n/a | 1. Subject headings: (((DE "Meditation") OR (DE "Mindfulness")) OR (DE "Mindfulness-Based Interventions")) OR (DE "Yoga") | 1. Subject headings: ((DE "Adult Offspring") OR (DE "Caregivers")) OR (DE "Family") |
|  | 1. Keyword Search (title only): "cancer of the lung*" OR "lung cancer*" OR "lung carcinoma*" OR "lung neoplasm*" OR "lung tumour*" OR "lung tumor*" OR nsclc OR "pulmonary carcinoma*" OR "pulmonary tumor*" OR "pulmonary tumour*" OR sclc | 1. Keyword search (title only): "breathing exercis*" OR MBCR OR MBCT OR MBSR OR meditat* OR mindful* OR "stress reduc*" OR yoga | 1. Keyword search (title only): "adult children" OR "care giver*" OR caregiver* OR "care giving" OR caregiving OR carer OR carers OR "care taker*" OR caretaker* OR caring OR couple* OR custodian* OR daughter* OR dependents OR dyad* OR families OR family OR folk* OR guardian* OR husband* OR kinship OR parent* OR partner* OR relatives OR sibling* OR son OR sons OR spous* OR wife OR wives |
|  | 1. Keyword search (abstract only): "cancer of the lung*" OR "lung cancer*" OR "lung carcinoma*" OR "lung neoplasm*" OR "lung tumour*" OR "lung tumor*" OR nsclc OR "pulmonary carcinoma*" OR "pulmonary tumor*" OR "pulmonary tumour*" OR sclc | 1. Keyword search (abstract only): "breathing exercis*" OR MBCR OR MBCT OR MBSR OR meditat* OR mindful* OR "stress reduc*" OR yoga | 1. Keyword search (abstract only): "adult children" OR "care giver*" OR caregiver* OR "care giving" OR caregiving OR carer OR carers OR "care taker*" OR caretaker* OR caring OR couple* OR custodian* OR daughter* OR dependents OR dyad* OR families OR family OR folk* OR guardian* OR husband* OR kinship OR parent* OR partner* OR relatives OR sibling* OR son OR sons OR spous* OR wife OR wives |
| Embase | 1. Subject heading: 'lung cancer'/exp | 1. Subject headings: 'breathing exercise'/de OR 'meditation'/exp OR 'mindfulness'/exp OR 'mindfulness meditation'/exp OR 'mindfulness based stress reduction'/exp OR 'stress reduction'/exp OR 'yoga'/exp | 1. Subject Headings: 'adult child'/exp OR 'caregiver'/exp OR 'family'/exp |
|  | 1. Keyword search (title and abstract): 'cancer of the lung':ti,ab OR 'lung cancer*':ti,ab OR 'lung carcinoma*':ti,ab OR 'lung neoplasm*':ti,ab OR 'lung tumour*':ti,ab OR 'lung tumor*':ti,ab OR nsclc:ti,ab OR 'pulmonary carcinoma*':ti,ab OR 'pulmonary tumour*':ti,ab OR 'pulmonary tumor*':ti,ab OR sclc:ti,ab | 1. Keyword search (title and abstract): 'breathing exercis*':ti,ab OR MBCR:ti,ab OR MBCT:ti,ab OR MBSR:ti,ab OR meditat*:ti,ab OR mindful*:ti,ab OR 'stress reduc*':ti,ab OR yoga:ti,ab | 1. Keyword search (title and abstract): 'adult children':ti,ab OR 'care giver*':ti,ab OR caregiver*:ti,ab OR 'care giving':ti,ab OR caregiving:ti,ab OR carer:ti,ab OR carers:ti,ab OR 'care taker*':ti,ab OR caretaker*:ti,ab OR caring:ti,ab OR couple*:ti,ab OR custodian*:ti,ab OR daughter*:ti,ab OR dependents:ti,ab OR dyad*:ti,ab OR families:ti,ab OR family:ti,ab OR folk*:ti,ab OR guardian*:ti,ab OR husband*:ti,ab OR kinship:ti,ab OR parent*:ti,ab OR partner*:ti,ab OR relatives:ti,ab ORsibling*:ti,ab OR son:ti,ab OR sons:ti,ab OR spous*:ti,ab OR wife:ti,ab OR wives:ti,ab |
| Cochrane CENTRAL | 1. Subject heading: [Lung Neoplasms] explode all trees MeSH | 1. Subject headings: Breathing Exercises [MeSH:NoExp] OR Meditation [MeSH] explode all tress OR Mindfulness [MeSH] explode all trees OR Stress, Psychological MeSH explode all trees with qualifier: psychology OR Stress, Psychological MeSH explode all trees with qualifier: therapy OR Yoga [MeSH] explode all trees | 1. Subject headings: Children [MeSH] explode all trees OR Caregivers [MeSH] explode all trees OR Family [MeSH] this term only |
|  | 1. Keyword search (title and abstract): "cancer of the lung":ti,ab OR lung NEXT cancer*:ti,ab OR lung NEXT carcinoma*:ti,ab OR lung NEXT neoplasm*:ti,ab OR lung NEXT tumour*:ti,ab OR lung NEXT tumor*:ti,ab OR nsclc:ti,ab OR pulmonary NEXT carcinoma*:ti,ab OR pulmonary NEXT tumor*:ti,ab OR pulmonary NEXT tumour*:ti,ab OR sclc:ti,ab | 1. Keyword search (title and abstract): breathing NEXT exercis*:ti,ab OR MBCR:ti,ab OR MBCT:ti,ab OR MBSR:ti,ab OR meditat*:ti,ab OR mindful*:ti,ab OR stress NEXT reduc*:ti,ab OR yoga:ti,ab | 1. Keyword search (title and abstract): "adult children":ti,ab OR care NEXT giver*:ti,ab OR caregiver*:ti,ab OR “care giving”:ti,ab OR caregiving:ti,ab OR carer:ti,ab OR carers:ti,ab OR care NEXT taker*:ti,ab OR caretaker*:ti,ab OR caring:ti,ab OR couple*:ti,ab OR custodian*:ti,ab OR daughter*:ti,ab OR dependents:ti,ab OR dyad*:ti,ab OR families:ti,ab OR family:ti,ab OR folk*:ti,ab OR guardian*:ti,ab OR husband*:ti,ab OR kinship:ti,ab OR parent*:ti,ab OR partner*:ti,ab OR relatives:ti,ab OR sibling*:ti,ab OR son:ti,ab OR sons:ti,ab OR spous*:ti,ab OR wife:ti,ab OR wives:ti,ab |
| *Notes*. The numbers in the table refer to the order in which each search was conducted within a given database. Some databases (e.g., CINAHL Complete) required specification of title versus abstract searches when inputting keywords, whereas others (e.g., PubMed) did not require this nomenclature. | | | |
